# The mediating role of healthy eating attitudes in the relationship between nutrition literacy and sustainable and healthy eating behaviors among young adults: a cross-sectional study

**DOI:** 10.3389/fpubh.2026.1885664

**Published:** 2026-07-08

**Authors:** Emre Duman, Serap Balaban-Barta

**Affiliations:** 1 Department of Nutrition and Dietetics, Faculty of Health Sciences, Siirt University, Siirt, Türkiye; 2 Department of Nutrition and Dietetics, Faculty of Health Sciences, Gaziantep University, Gaziantep, Türkiye

**Keywords:** food insecurity, healthy eating attitudes, mediation analysis, nutrition literacy, sustainable nutrition, young adults

## Abstract

**Background:**

This study was conducted to examine the associations of nutrition literacy, healthy eating attitudes, and sustainable and healthy eating behaviors with food insecurity among young adults, and to assess the mediating role of healthy eating attitudes in the relationship between nutrition literacy and sustainable and healthy eating behaviors.

**Methods:**

This cross-sectional study included 600 young adults aged 18–25 years from Siirt and Gaziantep, Türkiye. Data were collected using a sociodemographic form, Turkish Short Nutrition Literacy Scale for Young Adults (S-NutLit-Tr), Attitude Scale for Healthy Nutrition (ASHN), the Sustainable and Healthy Eating Behaviors Scale (SHE Behaviors), and the Food Insecurity Experience Scale (FIES). Group differences, predictors of SHE Behaviors, and the indirect association through healthy eating attitudes were examined using Kruskal-Wallis tests, multiple linear regression, and path analysis, respectively.

**Results:**

The mean age of the participants was 20.56 ± 1.87 years, and 50.7% were female. While 50.7% of the participants were food secure, moderate food insecurity was identified in 23.8% and severe food insecurity in 25.5%. Significant differences were observed across FIES groups in total S-NutLit-Tr and ASHN scores (Kruskal-Wallis H(2) = 8.752, *p* = 0.013; H(2) = 9.088, *p* = 0.011, respectively), while no significant difference was found in SHE Behaviors score (H(2) = 1.731, *p* = 0.421). In the multiple linear regression analysis, S-NutLit-Tr score (*β* = 0.470, *p* < 0.001), ASHN score (*β* = 0.177, *p* < 0.001), and sex (*β* = 0.083, *p* = 0.023) were significant predictors of the total SHE Behaviors score. In the indirect-effect model, the total effect of nutrition literacy on sustainable and healthy eating behaviors was significant. Healthy eating attitudes showed a small but statistically significant indirect effect, with an unstandardized coefficient (B) of 0.171 and a 95% confidence interval (CI) from 0.095 to 0.275. This indirect effect accounted for approximately 9% of the total effect.

**Conclusion:**

Among young adults, nutrition literacy and positive attitudes toward healthy eating are positively associated with sustainable and healthy eating behaviors. Healthy eating attitudes represented a small but statistically significant indirect pathway in this association. These findings suggest that interventions targeting young adults may benefit from addressing both the improvement of nutrition literacy and the strengthening of healthy eating attitudes.

## 1 Introduction

Young adulthood is a critical period marked by numerous life transitions, such as starting university education and moving away from the family home, which may have lasting effects on diet quality (1). During this period, young adults who begin university life have been reported to experience a decline in dietary quality, with time constraints, the high cost of healthy foods, and peer pressure emerging as major barriers to healthy eating and home meal preparation (2). Unfavorable changes in eating behaviors are commonly observed during the transition to young adulthood. In particular, lack of motivation, limited time, and low cooking self-efficacy are among the main barriers to maintaining healthy eating habits and preparing meals at home (2). In this context, nutrition literacy is considered an important factor in understanding young adults’ daily food choices (3, 4). Sustainable and healthy eating behaviors are conceptually defined as a multidimensional construct that includes behaviors such as meat reduction, avoiding food waste, choosing seasonal and local foods, and preferring quality-labeled products (5, 6). These behaviors are considered important not only for individual health, but also for population and planetary health. Indeed, the overlap between high diet quality and environmentally sustainable food choices offers combined benefits by contributing both to the reduction of disease burden and to lower greenhouse gas emissions (7, 8). Nevertheless, it has been noted that sustainable nutrition literacy needs to be examined more comprehensively across different sociocultural contexts (9). In Türkiye, evidence on the relationships among these variables remains limited, particularly in regions such as Southeastern Anatolia, where socioeconomic conditions and traditional dietary patterns play an important role.

Nutrition literacy is conceptualized as individuals’ capacity to obtain, understand, critically evaluate, and use food and nutrition information in their daily food choices, and it is recognized as one of the key skills associated with healthy eating behaviors (3, 4). Higher levels of nutrition literacy have been associated with greater fruit and vegetable consumption, a lower risk of obesity, and lower added sugar intake (3, 10). This association also appears to extend to the sustainability dimension. Kabasakal-Cetin et al. (5) reported that higher food literacy among young adults was associated with lower ultra-processed food consumption and greater sustainable and healthy eating patterns, while Mortaş et al. (6) found a significant positive association between nutrition literacy and sustainable eating behaviors. However, it has been emphasized that theoretical knowledge alone may not be sufficient to achieve sustainable and healthy eating goals, and that skills and attitudes should also be considered together (9). Within this framework, studies examining food literacy and sustainable and healthy eating behaviors together remain limited, and these associations need to be retested in different sociocultural contexts (5, 9).

Attitude may be considered an important intermediate step in the translation of nutrition literacy into behavior. According to the Theory of Planned Behavior, an individual’s attitude toward a behavior is one of the key factors influencing the intention to perform that behavior and, ultimately, the behavior itself. Therefore, attitudes toward healthy eating may play an important role in how nutrition knowledge and literacy are reflected in healthy and sustainable eating behaviors (11). In this field, Lai et al. (4) showed that nutrition literacy mediated the relationship between multilevel factors and healthy eating behavior among young adults, while Lai et al. (12) reported that a scenario-based online literacy intervention produced changes in both knowledge and behavior. However, the potential mediating role of attitudes toward healthy eating in the relationship between nutrition literacy and sustainable and healthy eating behaviors has not been directly tested in a young adult sample, and this gap has been clearly noted in the literature (9).

In addition to individual-level factors such as nutrition literacy and attitudes toward healthy eating, sustainable and healthy eating behaviors may also be influenced by sociodemographic and structural determinants. Sex is one such determinant, as previous studies suggest that women may be more likely to adopt sustainable dietary choices because of health- and environment-related concerns. However, it should also be noted that sex-based differences may vary depending on the cultural and social context (7, 8). Therefore, sex should be included as an independent predictor in models of sustainable and healthy eating. The second critical structural determinant is food insecurity. Among young adults and university students, food insecurity has been associated with lower diet quality, lower intake of whole grains, fruits, and raw vegetables, and higher levels of disordered eating attitudes (13–15). However, the relationship of food insecurity with nutrition literacy, healthy eating attitudes, and sustainable and healthy eating behaviors has not been sufficiently examined in an integrated manner. In particular, under conditions of economic constraint, some behaviors that appear sustainable, such as meat reduction or avoiding food waste, may reflect necessary economic coping strategies rather than intentional environmental choices. Therefore, the association between food insecurity and sustainable and healthy eating behaviors requires more detailed evaluation (13).

Within this framework, studies addressing nutrition literacy, healthy eating attitudes, sustainable and healthy eating behaviors, and food insecurity within an integrated model remain limited. In addition, the potential mediating role of healthy eating attitudes in the relationship between nutrition literacy and sustainable and healthy eating behaviors has not been directly tested in young adults.

Accordingly, the present study aimed to examine the associations among nutrition literacy, healthy eating attitudes, sustainable and healthy eating behaviors, and food insecurity among young adults living in the Southeastern Anatolia Region of Türkiye. In addition, the mediating role of healthy eating attitudes in the relationship between nutrition literacy and sustainable and healthy eating behaviors was assessed. The following hypotheses were tested: (H1) Higher nutrition literacy and more positive healthy eating attitudes are associated with higher sustainable and healthy eating behaviors. (H2) Healthy eating attitudes have a statistically significant indirect role in the association between nutrition literacy and sustainable and healthy eating behaviors. (H3) Nutrition literacy, healthy eating attitudes, and sustainable and healthy eating behaviors may vary according to food insecurity status.

## 2 Materials and methods

### 2.1 Study design and participants

This cross-sectional observational study was conducted to examine the associations among nutrition literacy, healthy eating attitudes, sustainable and healthy eating behaviors, and food insecurity among young adults aged 18–25 years living in two provinces of the Southeastern Anatolia Region of Türkiye, Siirt and Gaziantep. The study population consisted of young adults aged 18–25 years living in the central districts of Siirt and Gaziantep during the data collection period. Siirt and Gaziantep were selected because the research team had institutional and field access in these provinces, which made face-to-face data collection feasible within the planned study period. A convenience sampling approach was used for sample selection. Participants were recruited face-to-face from multiple public locations commonly frequented by young adults, including university campus areas, event venues, parks, and similar public settings. The study data were collected between February and March 2026.

The inclusion criteria were determined as being between 18 and 25 years of age, being able to read and understand Turkish, voluntarily agreeing to participate in the study, and providing informed consent. Individuals with cognitive or health conditions that prevented questionnaire completion, as well as those who did not meet the inclusion criteria, were excluded. The data were checked during face-to-face administration, and missing items were reviewed in the field. To minimize the possibility of duplicate participation, each questionnaire was administered once under direct researcher supervision. Before administration, potential participants were asked whether they had already completed the survey at another data collection location. Individuals who reported prior participation were not enrolled again. Data collection was conducted on scheduled days at each site within a short fieldwork period, which further reduced the likelihood of repeated participation. The dataset was also examined for missing data and outliers. The final sample consisted of 600 participants who provided analyzable data for the main study variables.

### 2.2 Ethical approval

The study was approved by the Siirt University Non-Interventional Clinical Research Ethics Committee (Date: January 30, 2026; Decision No.: 2026/01/02/5). The research was conducted in accordance with the principles of the Declaration of Helsinki. All participants were informed about the purpose of the study, that participation was voluntary, and that their responses would be evaluated anonymously and used only for scientific purposes. The data collection process began only after their written informed consent had been obtained.

### 2.3 Sample size and power analysis

The sample size of the study was determined through an *a priori* power analysis performed using G*Power 3.1.9.7 during the study planning phase. In the analysis conducted for the multiple linear regression model, a small effect size (f^2^ = 0.05), *α* = 0.05, statistical power (1 − *β*) = 0.90, and a total of six predictor variables were assumed. Under these assumptions, the minimum required sample size was calculated as 355. The final sample of 600 participants analyzed in the present study exceeded this minimum requirement. The value of 355 refers to the *a priori* minimum required sample size for the planned multiple linear regression model. It was not intended to represent a universal minimum for all statistical analyses conducted in the study. The regression model was used as the primary sample size criterion because it included the largest number of prespecified predictors among the main planned analyses. The achieved sample size of 600 therefore exceeded the planned minimum and provided statistical power above 0.90 under the assumptions specified above.

### 2.4 Measurement instruments

The study data were collected using a questionnaire consisting of five sections. The questionnaire consisted of a sociodemographic information form, the Turkish Short Nutrition Literacy Scale for Young Adults (S-NutLit-Tr), the Attitude Scale for Healthy Nutrition (ASHN), the Sustainable and Healthy Eating Behaviors Scale (SHE Behaviors), and the Food Insecurity Experience Scale (FIES).

#### 2.4.1 Sociodemographic form

This form collected information on participants’ age, sex, educational status, employment status, and perceived economic status. In addition, variables such as body weight, height, body mass index (BMI), marital status, presence of chronic disease, smoking status, and alcohol consumption were evaluated descriptively in the study findings. Body weight and height were obtained based on participants’ self-reports. BMI was calculated by dividing body weight in kilograms by the square of height in meters (kg/m^2^). Participants were classified according to the World Health Organization (WHO) BMI classification as underweight (<18.5 kg/m^2^), normal weight (18.5–24.99 kg/m^2^), overweight (25.0–29.99 kg/m^2^), or obesity (≥30.0 kg/m^2^) (16).

#### 2.4.2 Turkish short nutrition literacy scale for young adults (S-NutLit-Tr)

The scale was developed by Vrinten et al. (17), and its Turkish adaptation and validity–reliability study was conducted by Koc et al. (18). The scale consists of 11 items and is scored on a 5-point Likert-type scale. It includes two subscales: nutritional knowledge skills (items 1–8) and expert skills (items 9–11). Total scores range from 11 to 55, with higher scores indicating higher nutrition literacy. The total Cronbach’s *α* coefficient was reported as 0.80 in the original study and 0.86 in the Turkish adaptation study. In the present study, the Cronbach’s α coefficient of the scale was calculated as 0.93.

#### 2.4.3 Attitude scale for healthy nutrition

The scale was developed in Türkiye by Tekkurşun Demir and Cicioğlu (19). The scale consists of 21 items and four subscales: Information on Nutrition (IN), Emotion for Nutrition (EN), Positive Nutrition (PN), and Malnutrition (M). The items are rated on a 5-point Likert-type scale; positive items are scored from 1 to 5, whereas negative items are reverse-coded and scored from 5 to 1 (English item wording is provided in Supplementary Table S1). Total scores range from 21 to 105, with higher scores indicating more positive attitudes toward healthy eating. For score interpretation, 21 points indicate a very low level, 22–42 points a low level, 43–63 points a moderate level, 64–84 points a high level, and ≥85 points an ideally high level of healthy eating attitude. In the scale development study, the Cronbach’s *α* coefficient for the total scale was not separately reported; however, the internal consistency coefficients for the subscales were reported as 0.90 for IN, 0.84 for EN, 0.75 for PN, and 0.83 for M (19). In the present study, consistent with the original study, reliability was evaluated at the subscale level, and Cronbach’s *α* coefficients were calculated as 0.94, 0.82, 0.84, and 0.85, respectively.

#### 2.4.4 Sustainable and healthy eating behaviors

The scale was developed by Żakowska-Biemans et al. (20) and the original form consists of 34 items and 8 subscales, with items rated on a 7-point Likert-type scale ranging from 1 = never to 7 = always. The Turkish adaptation and psychometric evaluation were conducted by Köksal et al. (21). After the removal of items 26 and 27, a 32-item, 7-factor structure was obtained. The subscales of the Turkish form were reported as quality labels, seasonal food and avoiding food waste, animal welfare, meat reduction, healthy and balanced diet, local food, and low fat. In the present study, the scale score was evaluated based on the sum of item scores, with a total score range of 32–224. Higher scores indicate more favorable sustainable and healthy eating behaviors. The total Cronbach’s *α* coefficient was reported as 0.91 in the adaptation study. In the present study, the Cronbach’s α coefficient of the scale was calculated as 0.97.

#### 2.4.5 Food insecurity experience scale

The FIES is an experience-based 8-item scale developed as a global tool for monitoring food insecurity (22). The Turkish adaptation, validity, and reliability study of the scale was conducted by Kılınç et al. (23). The scale assesses food insecurity experiences over the past 12 months using yes/no responses. In the present study, FIES was analyzed both categorically for group comparisons and as a continuous total score in regression-based models. Total scores range from 0 to 8, with higher scores indicating more severe food insecurity. Scale scores are classified as food secure for 0–3 points, moderate food insecurity for 4–6 points, and severe food insecurity for 7–8 points (23). In the Turkish adaptation study, the Cronbach’s *α* coefficient was reported as 0.85. In the present study, the Cronbach’s α coefficient of the scale was calculated as 0.91.

### 2.5 Statistical analysis

The statistical analysis of data was performed using IBM SPSS Statistics for Windows, Version 27.0 (IBM Corp., Armonk, NY, USA) and AMOS Version 22.0 (IBM Corp., Armonk, NY, USA). Descriptive statistics are presented as mean ± standard deviation for continuous variables and as numbers and percentages for categorical variables. The internal consistency of the scales in the study sample was evaluated using Cronbach’s *α* coefficients. The normality of the distribution of continuous variables was assessed using the Kolmogorov–Smirnov and Shapiro–Wilk tests, along with skewness and kurtosis coefficients, an examination of histograms and Q–Q plots. The normality tests indicated that the scale scores did not meet the assumption of normal distribution in at least one group (*p* < 0.05). Levene’s test also indicated that the assumption of homogeneity of variance across groups was not satisfied (*p* < 0.05). Therefore, nonparametric tests were preferred for between-group comparisons. The statistically significant deviation of the raw variable distributions in the normality tests may result from the fact that even small deviations can reach statistical significance in large samples (*n* = 600). The assumptions critical for multiple linear regression and path analysis were therefore evaluated separately. The normal distribution of the residuals of the regression model was examined using a histogram and a P–P plot and was considered acceptable. Multicollinearity was assessed using the variance inflation factor (VIF) values and all VIF values ranged from 1.073 to 1.119 (tolerance: 0.893–0.932). The homogeneity of variance of the residuals was confirmed using a scatterplot of the standardized residuals against the standardized predicted values. The Durbin–Watson statistic was calculated as 1.833, and no autocorrelation was found in the residuals. Based on these evaluations, the use of parametric inferential models was considered appropriate. In addition, the bias-corrected bootstrap method used in the path analysis provides a robust estimation approach that is not dependent on distributional assumptions.

The relationships among the main study variables were examined using correlation analysis, and the strength of the associations was reported using the Spearman’s rho coefficient (*ρ*). To account for the distributional characteristics of Likert-type total scores, Spearman’s rank correlation was used in descriptive correlation analyses, whereas a parametric approach was applied in the inferential models, including regression and path analysis. The Kruskal-Wallis H test was used to compare total S-NutLit-Tr, ASHN, and SHE Behaviors scores according to food insecurity status. For significant omnibus test results, pairwise comparisons were performed using the Mann–Whitney U test. The Bonferroni method was applied to correct for multiple comparisons, with the adjusted significance threshold set at *p* < 0.017. Exploratory *post-hoc* analyses were also conducted to compare the seven SHE Behaviors subscale scores according to food insecurity status. Subscale scores were calculated as mean item scores. Group differences were examined using the Kruskal-Wallis H test. The Bonferroni adjusted significance threshold for the seven subscale comparisons was set at *p* < 0.0071. Pairwise Mann–Whitney U tests were planned only for subscales that met this adjusted omnibus threshold. Multiple linear regression analysis was performed to identify predictors of sustainable and healthy eating behaviors. In the model, the dependent variable was defined as the total SHE Behaviors score, and the independent variables were the total S-NutLit-Tr score, the total ASHN score, the total FIES score, age, sex, and BMI. Although the ASHN has a multidimensional structure, the total ASHN score was retained in the regression and mediation models because the scale provides an interpretable global score for healthy eating attitudes and established total score categories. This approach also preserved model parsimony. Internal consistency was reported at the subscale level to remain consistent with the original validation study. The regression results were reported with the coefficient (B), standard error, standardized beta (*β*), 95% confidence interval, and *p*-value. In addition, exploratory *post-hoc* analyses were performed for the seven SHE Behaviors subscales. Sex differences were first evaluated using the Mann–Whitney U test, with the Bonferroni adjusted significance threshold set at *p* < 0.0071 for seven subscale comparisons. As a sensitivity analysis, each subscale was also entered as the dependent variable in a separate linear regression model including S-NutLit-Tr, ASHN, FIES, age, sex, and BMI.

The mediating role of healthy eating attitudes in the relationship between nutrition literacy and sustainable and healthy eating behaviors was tested using a simple mediation model. The significance of the indirect effect was assessed using bias-corrected bootstrap confidence intervals with 5,000 resamples. The mediation model was specified using observed total scale scores. Nutrition literacy was entered as the independent variable, healthy eating attitudes as the mediator, and sustainable and healthy eating behaviors as the dependent variable. Age, sex, and BMI were entered as covariates predicting both the mediator and the outcome. All exogenous variables, namely nutrition literacy, age, sex, and BMI, were allowed to covary freely. The model included six observed variables. Therefore, the sample covariance matrix contained 21 non-redundant observed moments. The model included 21 free parameters. Accordingly, the model was just-identified, with 21 observed moments and 21 estimated parameters. The degrees of freedom were 0. The model reproduced the observed covariance matrix exactly. The chi-square value was 0.000, and the probability level could not be computed. Therefore, global fit indices were not reported because they are not informative in a just-identified model. Model evaluation focused on the estimated path coefficients and the bias-corrected bootstrap confidence interval for the indirect effect. Given the conceptual relevance of food insecurity, the indirect effect was re-estimated in a sensitivity model that additionally included the total FIES score. FIES was specified as an additional covariate predicting both ASHN and SHE Behaviors. FIES was also allowed to covary freely with the other exogenous variables. This sensitivity model was also just-identified, with 28 non-redundant observed moments and 28 freely estimated parameters. The degrees of freedom were 0. The results of this sensitivity model were compared with the main model adjusted for age, sex, and BMI. The unstandardized and standardized effects for the path coefficients and the bias-corrected 95% bootstrap confidence intervals were reported. In these analyses, *ρ*, standardized beta coefficients (*β*), and R^2^ values for model explanatory power were reported as effect size indicators.

## 3 Results

Table 1 presents the sociodemographic and baseline characteristics of the participants. The mean age of the participants was 20.56 ± 1.87 years. Women comprised 50.7% of the sample, and men accounted for 49.3%. Regarding marital status, most participants were single (97.3%). In terms of education, most participants had a university-level education (92.2%). Regarding employment status, 87.2% of the participants were unemployed. Half of the participants (50.0%) reported that their income was below their expenses. Anthropometric measurements showed a mean body weight of 67.44 ± 13.84 kg. The mean height was 171.65 ± 9.18 cm, and the mean BMI was 22.78 ± 3.69 kg/m^2^. Based on BMI classification, most participants were in the normal weight category (67.2%). This was followed by the overweight (18.5%), underweight (9.7%), and obesity (4.7%) groups. A total of 91.7% of the participants reported no physician-diagnosed chronic disease. Regarding lifestyle habits, 58.7% of the participants had never smoked, and 88.5% did not consume alcohol. The evaluation of food insecurity showed that 50.7% of the participants were food secure. Moderate food insecurity was observed in 23.8% of the sample, and severe food insecurity was present in 25.5%.

**Table 1:** Socio-demographic characteristics.

Variables	Value
Age (years), *mean ± SD*	20.56 ± 1.87
Body weight (kg), *mean ± SD*	67.44 ± 13.84
Height (cm), *mean ± SD*	171.65 ± 9.18
BMI (kg/m^2^), *mean ± SD*	22.78 ± 3.69
Sex, n (%)	
Female	304 (50.7)
Male	296 (49.3)
Marital status, *n (%)*	
Single	584 (97.3)
Married	16 (2.7)
Educational status, *n (%)*	
High school or below	42 (7.0)
University	553 (92.2)
Graduate/other	5 (0.8)
Employment status, *n (%)*	
Unemployed	523 (87.2)
Employed, full-time	63 (10.5)
Employed, part-time	14 (2.3)
Perceived economic status, *n (%)*	
Income lower than expenses	300 (50.0)
Income equal to expenses	246 (41.0)
Income higher than expenses	54 (9.0)
BMI category (WHO), *n (%)*	
Underweight	58 (9.7)
Normal weight	403 (67.2)
Overweight	111 (18.5)
Obesity	28 (4.7)
Physician-diagnosed chronic disease, *n (%)*	
No	550 (91.7)
Yes	50 (8.3)
Smoking status, *n (%)*	
Never	352 (58.7)
Former smoker	35 (5.8)
Current smoker	213 (35.5)
Alcohol consumption, *n (%)*	
None	531 (88.5)
Occasional	54 (9.0)
Regular	15 (2.5)
Food insecurity, *n (%)*	
Food secure	304 (50.7)
Moderate food insecurity	143 (23.8)
Severe food insecurity	153 (25.5)

BMI, Body Mass Index. Continuous variables are presented as mean ± SD, and categorical variables are presented as n (%).

Table 2 presents the summary statistics and internal consistency coefficients of the main scale scores. The mean S-NutLit-Tr score was 34.80 ± 10.01, with an observed range of 11.00 to 55.00. The mean ASHN score was 67.61 ± 9.68, with an observed range of 31.00 to 103.00. The mean SHE Behaviors score was 118.08 ± 37.13, and scores ranged from 32.00 to 224.00. The mean FIES score was 3.53 ± 3.05, with scores ranging from 0 to 8. The scales showed acceptable to high internal consistency reliability. The highest Cronbach’s *α* coefficient was observed for SHE Behaviors (0.97), followed by S-NutLit-Tr (0.93) and FIES (0.91). Given the multidimensional structure of ASHN, internal consistency was evaluated at the subscale level, in line with the original study. The Cronbach’s α coefficients for the subscales ranged from 0.82 to 0.94, and all values were above the acceptable level.

**Table 2:** Summary statistics and internal consistency coefficients of the main scale scores.

Scale	Number of items	Observed range	Mean ± SD	Cronbach’s α
S-NutLit-Tr	11	11.00–55.00	34.80 ± 10.01	0.93
ASHN^*^	21	31.00–103.00	67.61 ± 9.68	–
ASHN–IN	5	5.00–25.00	18.23 ± 5.29	0.94
ASHN–EN	6	6.00–30.00	17.22 ± 5.46	0.82
ASHN–PN	5	5.00–25.00	15.88 ± 4.81	0.84
ASHN–M	5	5.00–25.00	16.29 ± 4.98	0.85
SHE Behaviors	32	32.00–224.00	118.08 ± 37.13	0.97
FIES	8	0–8	3.53 ± 3.05	0.91

S-NutLit-Tr, Turkish Short Nutrition Literacy Scale for Young Adults; ASHN, Attitude Scale for Healthy Nutrition; SHE Behaviors, Sustainable and Healthy Eating Behaviors; FIES, Food Insecurity Experience Scale; IN, Information on Nutrition; EN, Emotion for Nutrition; PN, Positive Nutrition; M, Malnutrition. The original scale development study did not report an internal consistency coefficient for the total ASHN score. Given the multidimensional structure of the scale, reliability was evaluated at the subscale level in the present study.

Table 3 presents the comparison of total S-NutLit-Tr, ASHN, and SHE Behaviors scores according to food insecurity status. The Kruskal-Wallis test showed a statistically significant difference in total S-NutLit-Tr scores among the food insecurity groups (H (2) = 8.752, *p* = 0.013). Similarly, the total ASHN score differed across the groups (H (2) = 9.088, *p* = 0.011). In contrast, no statistically significant difference was found among the groups in the total SHE Behaviors score (H (2) = 1.731, *p* = 0.421). Pairwise comparisons with Bonferroni correction showed that the S-NutLit-Tr score was higher in the food-secure group than in the moderately food-insecure group (*p* = 0.008). The ASHN score was also higher in the food-secure group than in the severely food-insecure group (*p* = 0.002). Pairwise comparisons were not conducted for SHE Behaviors scores because the omnibus test was non-significant.

**Table 3:** Comparison of total S-NutLit-Tr, ASHN, and SHE Behaviors scores according to food insecurity status.

Variable	Food secure (*n* = 304)	Moderate food insecurity (*n* = 143)	Severe food insecurity (*n* = 153)	Kruskal-Wallis H statistic	*p*-value
S-NutLit-Tr, *median [Q1, Q3]*	36.0^a^ [32.0, 43.0]	33.0^b^ [27.0, 40.0]	33.0^ab^ [25.0, 43.0]	8.752	**0.013**
ASHN, *median [Q1, Q3]*	66.0^a^ [63.0, 75.0]	65.0^ab^ [61.0, 73.0]	64.0^b^ [62.0, 70.0]	9.088	**0.011**
SHE Behaviors, *median [Q1, Q3]*	120.5 [96.0, 142.0]	115.0 [91.0, 135.0]	121.0 [89.0, 142.0]	1.731	0.421

S-NutLit-Tr, Turkish Short Nutrition Literacy Scale for Young Adults; ASHN, Attitude Scale for Healthy Nutrition; SHE Behaviors, Sustainable and Healthy Eating Behaviors. Values are presented as median [Q1, Q3]. Group comparisons were evaluated using the Kruskal-Wallis H test. H denotes the Kruskal-Wallis test statistic. In this analysis, H is reported with 2 degrees of freedom because the comparisons involved three food-insecurity groups. Pairwise comparisons were conducted only when the omnibus test was statistically significant. Pairwise comparisons were performed using the Mann–Whitney U test on the same dataset, and the Bonferroni method was applied to correct for three pairwise comparisons. The adjusted significance threshold was set at *p* < 0.017. Groups with different superscript letters, a and b, differed statistically after Bonferroni correction. Pairwise comparisons were not conducted for SHE Behaviors because the omnibus test was non-significant. Bold *p*-values indicate statistically significant results.

Exploratory subscale-level analyses were performed to compare the seven SHE Behaviors subscale scores according to food insecurity status. There were no missing data for these analyses. None of the seven SHE Behaviors subscales differed significantly across food insecurity groups after Bonferroni correction. The lowest *p* value was observed for the meat reduction subscale, but this result did not meet the adjusted significance threshold. The detailed subscale results are presented in Supplementary Table S2.

Table 4 presents the multiple linear regression model predicting the total SHE Behaviors score. The regression model was statistically significant [*F*(6, 593) = 40.107, *p* < 0.001]. The independent variables explained 28.9% of the variance in the total SHE Behaviors score (R^2^ = 0.289, adjusted R^2^ = 0.281). The total S-NutLit-Tr score positively predicted the total SHE Behaviors score (B = 1.745, *β* = 0.470, *p* < 0.001). Similarly, the total ASHN score positively predicted the total SHE Behaviors score (B = 0.678, *β* = 0.177, *p* < 0.001). Regarding sex, male participants had higher total SHE Behaviors scores than female participants (B = 6.192, *β* = 0.083, *p* = 0.023). In contrast, the total FIES score, age, and BMI did not have statistically significant effects on the total SHE Behaviors score (*p* > 0.05). Among the variables tested, S-NutLit-Tr and ASHN were the only statistically significant scale-based predictors of SHE Behaviors.

**Table 4:** Multiple linear regression model predicting the total SHE Behaviors score.

Independent variable	B	SE	Std. *β*	95% CI	*p*-value
S-NutLit-Tr total score	1.745	0.135	0.470	1.479–2.010	**< 0.001**
ASHN total score	0.678	0.140	0.177	0.403–0.953	**< 0.001**
FIES total score	0.192	0.437	0.016	−0.666 – 1.049	0.661
Age	−0.213	0.718	−0.011	−1.623 – 1.196	0.766
Sex (0 = female, 1 = male)	6.192	2.719	0.083	0.852–11.532	**0.023**
BMI (kg/m^2^)	−0.329	0.361	−0.033	−1.038 – 0.380	0.362
Model summary			Value		
R^2^			0.289		
Adjusted R^2^			0.281		
F(6, 593)			40.107		
Model *p*			**< 0.001**		

The dependent variable was the total SHE Behaviors score. S-NutLit-Tr, Turkish Short Nutrition Literacy Scale for Young Adults; ASHN, Attitude Scale for Healthy Nutrition; FIES, Food Insecurity Experience Scale; BMI, Body Mass Index. B, unstandardized coefficient; SE, standard error; Std. β, standardized beta coefficient. CI, confidence interval. Bold p-values indicate statistically significant results.

Exploratory analyses were performed to examine sex-related patterns across the seven SHE Behaviors subscales. In the unadjusted Mann–Whitney U tests, no subscale differed significantly between female and male participants after Bonferroni correction. However, in the adjusted sensitivity models controlling for nutrition literacy, healthy eating attitudes, food insecurity, age, and BMI, male sex remained positively associated with the animal welfare subscale (B = 0.380, *β* = 0.138, *p* < 0.001) and the local food subscale (B = 0.304, *β* = 0.106, *p* = 0.007) after Bonferroni correction for the seven subscale comparisons. The associations for healthy and balanced diet (B = 0.217, *β* = 0.072, *p* = 0.049) and low fat (B = 0.228, *β* = 0.078, *p* = 0.042) reached the conventional *p* < 0.05 threshold before correction, but did not remain significant after correction for multiple comparisons. Therefore, these two findings were treated as exploratory and were not interpreted as robust associations. These exploratory findings suggest that the positive association between male sex and total SHE Behaviors may be partly related to selected subdomains rather than a uniform sex-related pattern across all components of the scale. The detailed subscale results are presented in Supplementary Table S3.

Table 5 presents the direct, indirect, and total effects of the simple mediation model involving nutrition literacy, attitudes toward healthy eating, and sustainable and healthy eating behaviors. The total S-NutLit-Tr score positively predicted the total ASHN score (B = 0.254, *β* = 0.262, *p* < 0.001). In addition, the total ASHN score positively predicted the total SHE Behaviors score (B = 0.675, *β* = 0.176, *p* < 0.001). Even after including ASHN as a mediator, the direct effect of S-NutLit-Tr on SHE Behaviors remained statistically significant (B = 1.739, *β* = 0.469, *p* < 0.001). Among the covariates, male sex was positively associated with SHE Behaviors (B = 6.376, *β* = 0.086, *p* = 0.017) but negatively associated with ASHN (B = −2.537, *β* = −0.131, *p* = 0.001). Age and BMI were not associated with either outcome (*p* > 0.05). In the mediation model, the predictors explained 9.6% of the variance in ASHN and 28.8% of the variance in SHE Behaviors. The effect decomposition showed that the total effect of S-NutLit-Tr on SHE Behaviors was statistically significant (B = 1.911, β = 0.515, *p* < 0.001). The indirect effect through ASHN was also statistically significant (B = 0.171, *β* = 0.046, 95% CI = 0.095 to 0.275). Because the 95% bias-corrected confidence interval did not include zero, the indirect association of S-NutLit-Tr with SHE Behaviors through ASHN was statistically significant. The indirect effect accounted for approximately 9% of the total effect. In the FIES-adjusted sensitivity model, the indirect association of S-NutLit-Tr with SHE Behaviors through ASHN remained statistically significant. The unstandardized indirect effect was B = 0.168, with a standardized estimate of *β* = 0.045 and a 95% bias-corrected bootstrap confidence interval from 0.091 to 0.270. This estimate was very similar to the main model indirect effect of B = 0.171, *β* = 0.046, with a 95% confidence interval from 0.095 to 0.275. The direct effect of S-NutLit-Tr on SHE Behaviors was also retained in the sensitivity model, B = 1.745, *β* = 0.470, with a 95% confidence interval from 1.381 to 2.054. The total effect was B = 1.913, *β* = 0.515, with a 95% confidence interval from 1.572 to 2.211. These findings indicate that the indirect association was robust to additional adjustment for food insecurity. A side-by-side comparison of the main model and the FIES-adjusted sensitivity model is presented in Supplementary Table S4.

**Table 5:** Direct, indirect, and total effects in the mediation model linking S-NutLit-Tr, ASHN, and SHE Behaviors.

Path / Effect	B	SE	Std. β	C. R.	*p*-value	BC 95% CI
Panel A. Structural paths						
S-NutLit-Tr → ASHN (a)	0.254	0.038	0.262	6.681	**< 0.001**	[0.181, 0.335]
Age → ASHN	0.283	0.208	0.055	1.362	0.173	[−0.142, 0.693]
Sex (0 = female, 1 = male) → ASHN	−2.537	0.779	−0.131	−3.258	**0.001**	[−4.044, −1.010]
BMI → ASHN	0.058	0.106	0.022	0.546	0.585	[−0.160, 0.268]
ASHN → SHE Behaviors (b)	0.675	0.139	0.176	4.852	**< 0.001**	[0.380, 0.963]
S-NutLit-Tr → SHE Behaviors (c′)	1.739	0.134	0.469	12.989	**< 0.001**	[1.376, 2.049]
Age → SHE Behaviors	−0.171	0.708	−0.009	−0.242	0.809	[−1.598, 1.315]
Sex (0 = female, 1 = male) → SHE Behaviors	6.376	2.674	0.086	2.385	**0.017**	[1.175, 11.801]
BMI → SHE Behaviors	−0.333	0.359	−0.033	−0.927	0.354	[−0.987, 0.353]
Panel B. Effect decomposition for S-NutLit-Tr on SHE Behaviors						
Total effect (c)	1.911	—	0.515	—	**< 0.001**	[1.571, 2.205]
Direct effect (c′)	1.739	—	0.469	—	**< 0.001**	[1.376, 2.049]
Indirect effect (a × b)	0.171	—	0.046	—	**< 0.001**	[0.095, 0.275]

S-NutLit-Tr, Turkish Short Nutrition Literacy Scale for Young Adults; ASHN, Attitude Scale for Healthy Nutrition; SHE Behaviors, Sustainable and Healthy Eating Behaviors; BMI, body mass index; BC, bias-corrected confidence interval; C. R., critical ratio, calculated as C. R. = B / SE, where B denotes the unstandardized estimate and SE denotes the standard error. It is interpreted as a z statistic. Notes: The model examined the indirect association of S-NutLit-Tr with SHE Behaviors through ASHN, adjusted for age, sex, and BMI. Sex was coded 0 = female and 1 = male. Unstandardized coefficients, standardized coefficients, and bias-corrected 95% bootstrap confidence intervals are shown. Squared multiple correlations were 0.096 for ASHN and 0.288 for SHE Behaviors. The indirect effect of S-NutLit-Tr on SHE Behaviors through ASHN was statistically significant because the 95% BC confidence interval did not include zero. The indirect effect accounted for approximately 9% of the total effect. The model was just-identified because the 21 non-redundant observed moments were matched by 21 freely estimated parameters. The degrees of freedom were therefore 0. The chi-square value was 0.000, and global fit indices were not informative. Bold *p*-values indicate statistically significant results.

**Figure 1:**
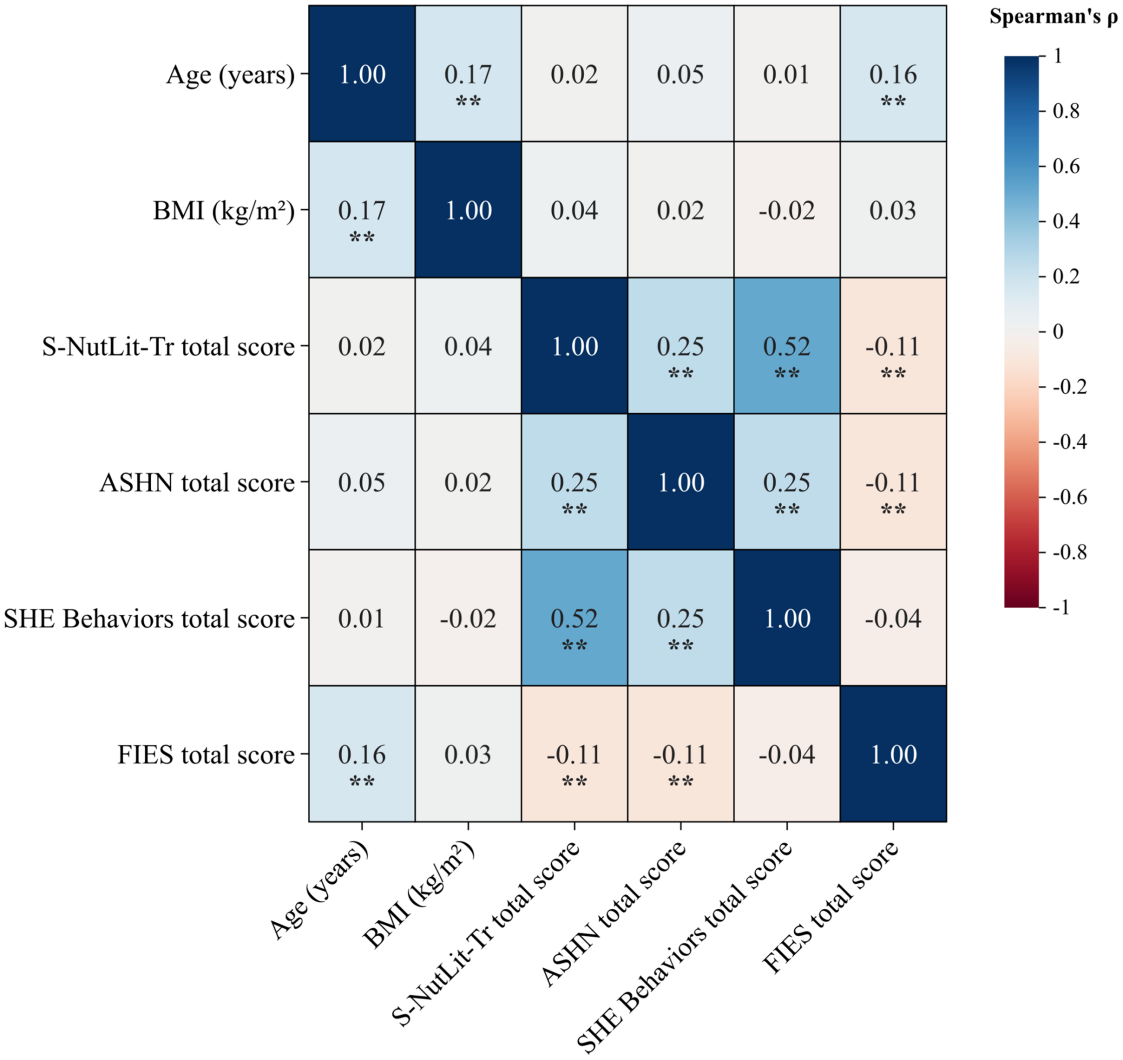
Spearman correlation heatmap of the main study variables. Cells display Spearman’s rho coefficients. The symbol ρ denotes Spearman’s rho correlation coefficient estimated from the sample data. The sign and magnitude of each correlation are also provided numerically within the cells. ** indicates *p* < 0.01 (two-tailed). The heatmap includes age, BMI, S-NutLit-Tr total score, ASHN total score, SHE Behaviors total score, and FIES total score. Correlation magnitude was interpreted using absolute values as follows: 0.00 to 0.19, very weak; 0.20 to 0.39, weak; 0.40 to 0.59, moderate; 0.60 to 0.79, strong; and 0.80 or higher, very strong. S-NutLit-Tr, Turkish Short Nutrition Literacy Scale for Young Adults; ASHN, Attitude Scale for Healthy Nutrition; SHE Behaviors, Sustainable and Healthy Eating Behaviors; FIES, Food Insecurity Experience Scale.

Figure 1 presents the correlations among the main study variables. Spearman correlations showed a moderate positive relationship between the total S-NutLit-Tr score and the total SHE Behaviors score (*ρ* = 0.52, *p* < 0.01). Positive correlations were also found between the total S-NutLit-Tr score and the total ASHN score (ρ = 0.25, *p* < 0.01), and between the total ASHN score and the total SHE Behaviors score (ρ = 0.25, *p* < 0.01). The total FIES score showed very weak negative correlations with the total S-NutLit-Tr and ASHN scores (ρ = −0.11, *p* < 0.01 for both). No statistically significant correlation was found between the total FIES score and the total SHE Behaviors score (ρ = −0.04, *p* > 0.05). Very weak positive correlations were also found between age and BMI (ρ = 0.17, *p* < 0.01), and between age and the total FIES score (ρ = 0.16, *p* < 0.01).

**Figure 2:**
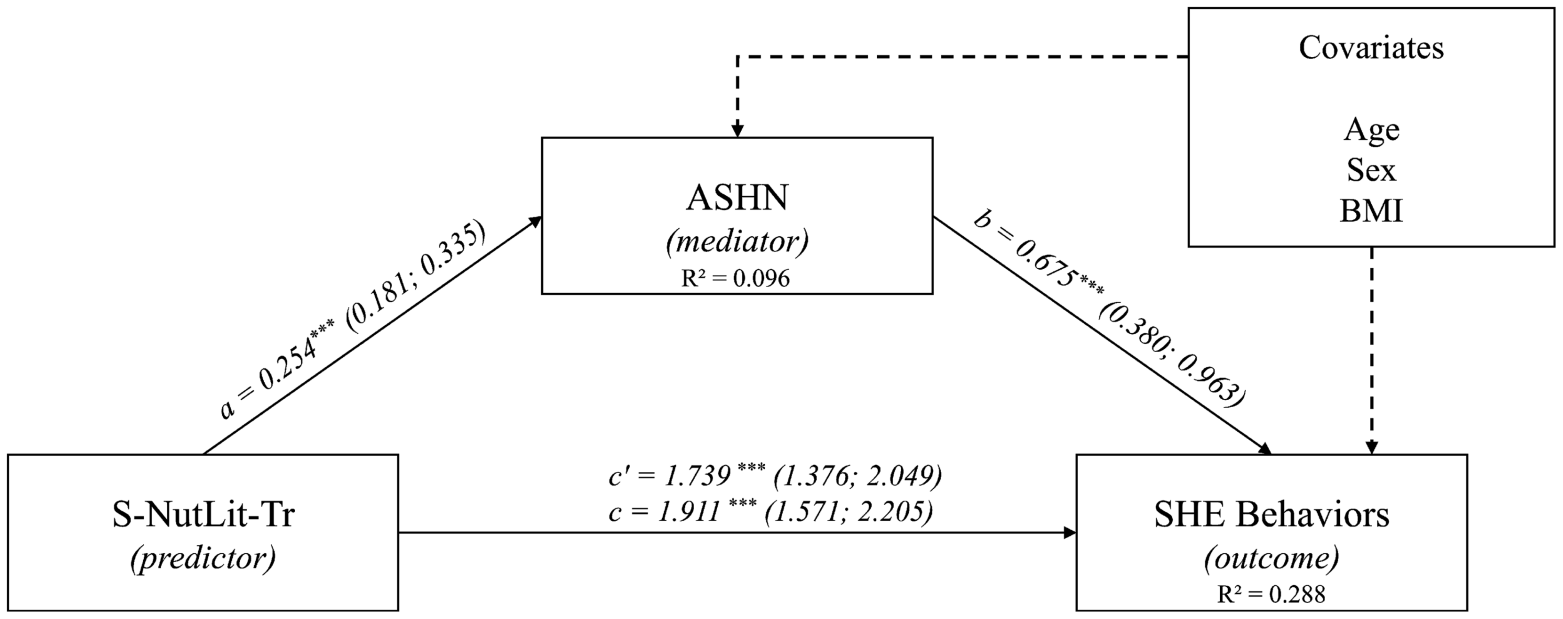
Cross-sectional simple mediation model examining the indirect association of nutrition literacy with sustainable and healthy eating behaviors via healthy eating attitudes. Values on the paths are unstandardized coefficients (B). Values in parentheses indicate 95% bias-corrected bootstrap confidence intervals. The total effect (c) and direct effect (c′) are both shown for clarity. Covariates (age, sex, and BMI) were included in the model but are not shown with individual coefficients to preserve figure readability. For visual clarity, the freely estimated covariances among the exogenous variables are not displayed in the figure. These covariances were included in the AMOS model specification. ***p < 0.001. S-NutLit-Tr, Turkish Short Nutrition Literacy Scale for Young Adults; ASHN, Attitude Scale for Healthy Nutrition; SHE Behaviors, Sustainable and Healthy Eating Behaviors.

Figure 2 illustrates the simple indirect-effect model summarized in Table 5. The model showed a positive path from S-NutLit-Tr to ASHN and from ASHN to SHE Behaviors. The predictors explained 9.6% of the variance in ASHN (R^2^ = 0.096) and 28.8% of the variance in SHE Behaviors (R^2^ = 0.288). The direct path from S-NutLit-Tr to SHE Behaviors remained statistically significant after ASHN was included in the model. Together, these paths indicate a statistically significant indirect association of S-NutLit-Tr with SHE Behaviors through ASHN.

## 4 Discussion

In this cross-sectional study, we examined the associations among nutrition literacy, healthy eating attitudes, sustainable and healthy eating behaviors, and food insecurity among young adults living in the Southeastern Anatolia Region of Türkiye. In the multiple linear regression analysis, total S-NutLit-Tr (*β* = 0.470, *p* < 0.001) and ASHN (*β* = 0.177, *p* < 0.001) scores were the two strongest predictors of SHE Behaviors, while sex made a small but statistically significant independent contribution to SHE Behaviors (males; *β* = 0.083, *p* = 0.023; Table 4). In the indirect-effect model tested with bias-corrected bootstrap confidence intervals, there was a small but statistically significant indirect association between nutrition literacy and sustainable and healthy eating behaviors through healthy eating attitudes (B = 0.171; 95% CI: 0.095–0.275; Table 5). In comparisons across food insecurity groups, significant differences were observed in S-NutLit-Tr (*p* = 0.013) and ASHN (*p* = 0.011) total scores, whereas no statistically significant difference was found in SHE Behaviors total scores (*p* = 0.421; Table 3). These findings suggest that nutrition literacy is a strong individual-level correlate of sustainable and healthy eating behaviors among young adults, while positive attitudes toward healthy eating may serve as an additional indirect pathway in this relationship.

Our findings indicate that young adults with higher nutrition literacy had more favorable sustainable and healthy eating behaviors. This result is consistent with previous studies. Mortaş et al. (6) reported that sustainable eating behaviors increased as nutrition literacy increased among young adults in Türkiye. Similarly, Kabasakal-Cetin et al. (5) showed that higher food literacy was associated with lower ultra-processed food consumption and more favorable sustainable and healthy eating behaviors among Turkish university students. Consistent with these findings, Helvacı et al. (24) reported that, in a sample of 319 Turkish young adults, sustainable food literacy was significantly predicted by female sex and positive eating attitudes. Similarly, in a recent study, Ünal et al. (25) found that each one-unit increase in the “reading comprehension and interpretation” subscale of nutrition literacy in adults was associated with a 4.4-unit increase in the SHE Behaviors score. Together, these findings support that nutrition literacy and healthy eating attitudes may be closely related to sustainable eating behaviors in the Turkish context. International findings also support this association: Lai et al. (4) reported that nutrition literacy made a significant contribution to healthy eating behavior beyond multilevel factors among university students in Taiwan; Bedoyan et al. (3) found that critical nutrition literacy was associated with lower dietary added sugar intake; and Yoo et al. (10) reported that higher food literacy was associated with higher fruit and vegetable consumption and lower obesity risk in an adult sample in Seoul. Sahadeo et al. (26) showed that awareness of sustainable food systems was low among South African university students. The fact that a substantial proportion of students were not familiar with the Sustainable Development Goals suggests that affordability, rather than environmental concern, may be a stronger determinant of food choices. Therefore, improving nutrition literacy may be an important component of promoting sustainable food choices. The contribution of the present study to this literature is that the relationship between nutrition literacy and sustainable and healthy eating behaviors was also demonstrated in a sample from the Southeastern Anatolia Region. This finding suggests that nutrition literacy is an important individual resource that may support not only healthy eating behaviors but also sustainable eating behaviors. In addition, the fact that the model explained approximately 29% of the variance in SHE Behaviors total scores, with nutrition literacy being the strongest predictor, indicates that this relationship is meaningful from a public health perspective.

The model indicated a statistically significant indirect pathway from nutrition literacy to sustainable and healthy eating behaviors through healthy eating attitudes. This finding suggests that nutrition literacy is directly associated with sustainable and healthy eating behaviors and may also be indirectly related to these behaviors through more favorable attitudes toward healthy eating. Çiğdem and Emre (27) reported a positive association between nutrition literacy and healthy eating attitudes among university students. Helvacı et al. (24) showed that healthy eating attitudes positively predicted sustainable food literacy among Turkish young adults. Mortaş et al. (6) also reported positive associations between food and nutrition literacy, including its attitude subdimension, and sustainable and healthy eating behaviors among young adults in Türkiye. Jiang et al. (28), in a structural equation modeling study among Chinese adolescents, showed that healthy eating knowledge had a direct effect on healthy eating practices and an indirect effect through attitudes. These findings suggest that healthy eating attitudes may represent one psychosocial component of this indirect pathway.

Although the indirect association observed in the present study was statistically significant, it was small, and the direct association between nutrition literacy and sustainable and healthy eating behaviors remained significant after healthy eating attitudes were included in the model. This finding indicates that the association between nutrition literacy and sustainable and healthy eating behaviors cannot be explained by attitude alone. Lai et al. (4), using nutrition literacy as the mediator, showed that nutrition literacy mediated the effects of several multilevel determinants, including healthy eating attitudes, preference for healthy food, and healthy eating self-efficacy, on healthy eating behavior among college students. Although the direction of mediation in that study differs from the present model, the findings indicate that the relationships among nutrition literacy, attitudes, and eating behavior are not limited to a simple knowledge-to-behavior pathway. Jurado-Gonzalez et al. (2) showed that lack of motivation, lack of time, and limited culinary knowledge were major barriers to healthy eating and home meal preparation among university students. Pandey et al. (29), in a Theory of Planned Behavior-based study, reported that attitude and perceived behavioral control predicted intention to consume plant-based yogurt alternatives, and that intention predicted consumption behavior. Jylhä et al. (30) found that attitudes were the strongest predictor of climate-friendly food-choice intentions among emerging adults, while attitudinal ambivalence weakened the association between attitudes and intentions. These findings are consistent with the indirect-effect pattern observed in the present study. Healthy eating attitudes appear to be one relevant indirect pathway, but self-efficacy, perceived behavioral control, motivation, time constraints, the food environment, economic accessibility, and cultural dietary norms may also contribute to sustainable and healthy eating behaviors.

One of the important findings of this study was that food insecurity groups differed significantly in terms of nutrition literacy and healthy eating attitudes, while no similar difference was observed in sustainable and healthy eating behaviors. At first glance, this finding may appear inconsistent with previous studies that have associated food insecurity with lower diet quality and unfavorable nutritional indicators. For example, Kim et al. (13) showed that the co-occurrence of low income and food insecurity was associated with poor diet and with indicators of anxiety and depression. Shi et al. (15) showed that, among Australian university students with food insecurity, diet quality and whole-grain consumption were lower and sugar consumption was higher. Slotnick et al. (31) reported that U. S. university students experiencing persistent food insecurity had 17% lower fruit consumption and 6% lower fiber intake, while their added sugar intake from sugar-sweetened beverages was 10% higher. Similarly, Memiç-İnan and Çapraz (14) showed that food insecurity among Turkish university students was associated with lower fruit and raw vegetable consumption and higher disordered eating attitude scores. However, the absence of a significant difference in total SHE Behaviors scores across food insecurity groups in the present study should be interpreted cautiously in light of the multidimensional structure of the scale. The SHE Behaviors scale evaluates several behavioral domains together, including meat reduction, seasonal food and avoiding food waste, local food, quality labels, and low fat. Some of these behaviors may reflect intentional environmental choices, whereas others may emerge as a consequence of economic constraints. For instance, behaviors such as meat reduction or avoiding food waste may result from limited purchasing power rather than sustainability awareness in some individuals. In contrast, the preference for quality-labeled or specialty products may be lower among individuals facing economic constraints. Therefore, opposing effects across different subdimensions of the scale may be considered a plausible explanation for the absence of a significant group difference in the total SHE Behaviors score, but this interpretation should be treated cautiously (20, 21). As also emphasized in a recent review (32), the relationship between socioeconomic conditions and sustainable eating behaviors is not always linear. Within this framework, we explored whether the non-significant total SHE Behaviors finding reflected opposite patterns across the heterogeneous subdimensions of the scale. However, the exploratory subscale-level analyses did not show statistically significant group differences after correction for multiple comparisons. In other words, while individuals facing economic constraints may consume fewer quality-labeled or specialty products, behaviors such as avoiding food waste, choosing seasonal products, or consuming certain expensive foods less frequently may emerge as a result of forced economic adaptation (33). Indeed, the finding that the FIES score was very weakly negatively associated with the S-NutLit-Tr (*ρ* = −0.11) and ASHN (ρ = −0.11) scores but did not show a significant correlation with the SHE Behaviors score is consistent with the possibility that food insecurity may relate differently to attitude, literacy, and behavior indicators. When some seemingly sustainable behaviors arise out of necessity in low-income individuals, the structural validity of the scale should be reevaluated at the subscale level in young adult samples (14). Therefore, when interpreting the SHE Behaviors scores, researchers should consider that some sustainable behaviors may reflect economic constraints rather than environmental awareness. Future studies should assess voluntary, environmentally motivated sustainable behaviors separately from those that arise out of economic necessity.

In the multiple linear regression analysis, being male significantly increased total SHE Behaviors scores (B = 6.192, *β* = 0.083, *p* = 0.023), whereas in the mediation model, the same variable had a significant negative effect on healthy eating attitudes (B = −2.537). This finding suggests that men may report higher levels of selected sustainable and healthy eating behaviors, although this pattern does not appear to be explained by more positive healthy eating attitudes. These results do not directly align with studies reporting stronger health-oriented or sustainability-related dietary tendencies among women (7, 24). However, Chard et al. (34) showed that the determinants of sustainable dietary intentions may vary by sex and cultural context. In the present study, unadjusted subscale comparisons did not show statistically significant sex differences after Bonferroni correction. However, the adjusted sensitivity models suggested that the positive total-score association for male sex may be partly attributable to selected subdomains, particularly animal welfare and local food. In contrast, the associations for healthy and balanced diet and low fat reached the conventional *p* < 0.05 threshold before correction, but did not remain significant after correction for multiple comparisons. These findings should therefore be interpreted as exploratory rather than robust sex-related differences. This pattern may help explain why male sex was positively associated with total SHE Behaviors despite being negatively associated with healthy eating attitudes. The SHE Behaviors total score combines heterogeneous domains, and some endorsed behaviors may reflect local food practices, availability, routine purchasing patterns, economic considerations, or other contextual motives rather than explicit health-oriented attitudes or intentional environmental concern. Therefore, these exploratory sex-related findings should be interpreted cautiously and should be examined in future studies using domain-specific models and measures that distinguish intentional sustainability motives from behaviors endorsed for other reasons.

### 4.1 Strengths and limitations

This study has several limitations. Its cross-sectional design allowed the associations among variables to be evaluated, but causal inference cannot be made. Although the tested model was specified from nutrition literacy to healthy eating attitudes and then to sustainable and healthy eating behaviors, the temporal order among these variables could not be definitively established. Therefore, the findings should be interpreted as a cross-sectional statistical indirect association rather than evidence of causal mediation. Moreover, the model explained only 9.6% of the variance in healthy eating attitudes, indicating that determinants such as peer influence, family role modeling, media exposure, previous dietary interventions, and personal eating experiences were not captured in the present study.

The sample was recruited through convenience sampling from the provinces of Siirt and Gaziantep in the Southeastern Anatolia Region. Place of residence, defined as urban or rural residence, was not recorded. Because recruitment was conducted mainly in the central districts of Siirt and Gaziantep, rural young adults are likely to be underrepresented. Residual confounding by urbanicity therefore cannot be excluded. Future studies should record place of residence and examine whether the observed associations differ between urban and rural young adults. In addition, the sample was predominantly single, university educated, and unemployed. Therefore, the findings may not be generalizable to all young adults, particularly non-university attending young adults, vocational trainees, rural non-university youth, full-time employed or early career young adults, and married or parenting young adults.

Body weight and height were obtained through self-report, which may have introduced measurement error in BMI estimation. Because young adults may underreport body weight or overreport height, BMI misclassification may have attenuated the associations between BMI and the main study variables. The use of self-reported scales may also have increased the possibility of social desirability bias, recall bias, and common method bias. This issue may be particularly relevant to the indirect-effect model, because nutrition literacy, healthy eating attitudes, and sustainable and healthy eating behaviors were all assessed by self-report questionnaires at the same time point.

Because the ASHN has a multidimensional structure, and the original validation study did not report a total scale internal consistency coefficient, reliability was evaluated at the subscale level in the present study. Although the total ASHN score was retained in the regression and mediation models as a global indicator of healthy eating attitudes, pooling four content-diverse subscales may have obscured domain-specific associations. Therefore, future studies should examine whether specific ASHN subscales, such as information on nutrition, emotion for nutrition, positive nutrition, or malnutrition, play different roles in the relationship between nutrition literacy and sustainable and healthy eating behaviors. Another measurement-related limitation concerns the use of the SHE Behaviors scale in food-insecure young adults. The scale assesses several sustainable and healthy eating domains, but it may not fully distinguish voluntary, environmentally motivated behaviors from behaviors that arise because of economic constraint. To our knowledge, the scale has not been specifically validated in food-insecure populations. Therefore, future studies should evaluate the structural validity and subscale performance of the SHE Behaviors scale in food-insecure samples. The present study also relied on self-reported behavioral tendencies rather than measured dietary intake, food purchasing data, biomarkers, or objective environmental impact indicators. Thus, SHE Behaviors scores may not directly correspond to objective dietary quality or actual environmental sustainability. Unmeasured variables related to income status, living arrangements, food environment, and lifestyle may also have resulted in residual confounding.

Another limitation concerns the mismatch in measurement time frames. The FIES captures food insecurity experiences over the past 12 months, whereas the S-NutLit-Tr, ASHN, and SHE Behaviors scales assess more current tendencies and behaviors. This temporal mismatch may have attenuated the observed associations and may partly explain why the FIES score was not significantly associated with the total SHE Behaviors score. Because the present study used a single-wave cross-sectional design, the stability of food insecurity patterns over time could not be determined. Therefore, sensitivity analyses restricted to participants with stable food insecurity trajectories were not feasible. Longitudinal studies using time-aligned measurement windows are needed to clarify the temporal relationships among food insecurity, nutrition literacy, healthy eating attitudes, and sustainable and healthy eating behaviors. Nevertheless, the relatively large sample size, the use of validated measurement tools, and the evaluation of an indirect-effect model are among the important strengths of the study.

## 5 Conclusion

This study showed that nutrition literacy and positive healthy eating attitudes were positively associated with sustainable and healthy eating behaviors among young adults. Nutrition literacy was one of the strongest predictors of sustainable and healthy eating behaviors. The statistical indirect-effect model showed that healthy eating attitudes had a small but statistically significant indirect role in this association, accounting for approximately 9% of the total effect. In contrast, no significant difference was found between food insecurity groups in terms of total sustainable and healthy eating behaviors. Therefore, nutrition interventions targeting young adults should adopt integrated approaches that improve nutrition literacy, support positive healthy eating attitudes, and consider economic accessibility. Future longitudinal studies with more representative samples from different regions are needed.

## Data Availability

The original contributions presented in the study are included in the article/Supplementary material, further inquiries can be directed to the corresponding author.
