# Supplementary material for: The mediating role of healthy eating attitudes in the relationship between nutrition literacy and sustainable and healthy eating behaviors among young adults: a cross-sectional study
Source: Front Public Health. 2026 Jul 8;14:1885664. doi: 10.3389/fpubh.2026.1885664 (PMC13388279; doi:10.3389/fpubh.2026.1885664)
Supplement: Supplementary file 1 [file Table_1.DOCX]

**Supplementary Table S1.** English Rendering of the Turkish Attitude Scale for Healthy Nutrition (ASHN)

| **IMPORTANT NOTE.** The original instrument was developed in Turkish in Türkiye. The Turkish source version should be regarded as the authoritative version for administration, scoring, and citation. The English wording below was prepared by the authors as an illustrative translation to help international readers, editors, and reviewers understand the item content. It is provided for transparency and comprehension only and should not be interpreted as a separately validated cross-cultural English version. |
| --- |

| Item | English item wording | Subscale | Scoring |
| --- | --- | --- | --- |
| 1 | I know the benefits of healthy eating. | Information on Nutrition | Direct |
| 2 | I know which foods contain protein. | Information on Nutrition | Direct |
| 3 | I know which foods contain carbohydrates. | Information on Nutrition | Direct |
| 4 | I know which foods contain vitamins/minerals. | Information on Nutrition | Direct |
| 5 | I know what healthy foods are. | Information on Nutrition | Direct |
| 6 | I feel happy when I consume sugary foods (e.g., chocolate, cake, biscuits). | Emotion for Nutrition | Reverse |
| 7 | I enjoy eating fast-food products (e.g., hamburgers, pizza). | Emotion for Nutrition | Reverse |
| 8 | I enjoy eating processed meat products (e.g., salami, sausages, sucuk). | Emotion for Nutrition | Reverse |
| 9 | I like eating deep-fried foods. | Emotion for Nutrition | Reverse |
| 10 | I do not like eating fruit. | Emotion for Nutrition | Reverse |
| 11 | I feel happy when I consume syrupy desserts (e.g., baklava, kunefe). | Emotion for Nutrition | Reverse |
| 12 | I eat main meals (breakfast, lunch, and dinner) regularly. | Positive Nutrition | Direct |
| 13 | I drink at least 1.5 L of water per day. | Positive Nutrition | Direct |
| 14 | I consume vegetables in at least three meals per week. | Positive Nutrition | Direct |
| 15 | I consume fruit regularly. | Positive Nutrition | Direct |
| 16 | I eat protein-containing foods (e.g., meat, milk, eggs) every day. | Positive Nutrition | Direct |
| 17 | I skip main meals. | Malnutrition | Reverse |
| 18 | I eat junk food (e.g., chips, chocolate, biscuits) every day. | Malnutrition | Reverse |
| 19 | I drink at least one glass of carbonated/fizzy beverages every day. | Malnutrition | Reverse |
| 20 | I eat on the go. | Malnutrition | Reverse |
| 21 | I usually replace a main meal with foods such as cake or biscuits. | Malnutrition | Reverse |

**Notes:**

• Response format: 5-point Likert scale (1 = Strongly disagree, 2 = Disagree, 3 = Undecided, 4 = Agree, 5 = Strongly agree).

• Direct-coded items: 1-5 and 12-16. Reverse-coded items: 6-11 and 17-21.

• The scale contains 21 items across four subscales: Information on Nutrition (items 1-5), Emotion for Nutrition (items 6-11), Positive Nutrition (items 12-16), and Malnutrition (items 17-21).

• Total score range: 21-105. Higher scores indicate a more positive attitude toward healthy nutrition.

**Reference:** Tekkurşun Demir G, Cicioğlu Hİ. Attitude Scale for Healthy Nutrition (ASHN): Validity and Reliability Study. Gaziantep University Journal of Sport Science (2019) 4:256-274. doi: 10.31680/gaunjss.559462
