# Supplementary material for: The mediating role of healthy eating attitudes in the relationship between nutrition literacy and sustainable and healthy eating behaviors among young adults: a cross-sectional study
Source: Front Public Health. 2026 Jul 8;14:1885664. doi: 10.3389/fpubh.2026.1885664 (PMC13388279; doi:10.3389/fpubh.2026.1885664)
Supplement: Supplementary file 2 [file Table_2.DOCX]

**Supplementary Table S2.** SHE Behaviors subscale scores according to food insecurity status

| SHE Behaviors subscale | Food secure  (n = 304) | Moderate food insecurity  (n = 143) | Severe food insecurity  (n = 153) | Kruskal-Wallis H | *p* |
| --- | --- | --- | --- | --- | --- |
| Quality labels | 3.50 [2.88, 4.25] | 3.38 [2.75, 4.13] | 3.25 [2.25, 4.38] | 3.629 | 0.163 |
| Seasonal food and avoiding food waste | 3.86 [3.00, 4.71] | 4.00 [2.86, 4.57] | 4.00 [2.93, 5.00] | 0.906 | 0.636 |
| Healthy and balanced diet | 4.00 [3.00, 5.25] | 4.00 [3.00, 5.00] | 4.00 [2.75, 5.00] | 1.684 | 0.431 |
| Local food | 3.00 [2.67, 4.00] | 3.00 [2.33, 4.00] | 3.67 [2.33, 4.67] | 4.165 | 0.125 |
| Meat reduction | 3.00 [2.33, 4.00] | 3.00 [2.00, 4.00] | 3.00 [2.00, 4.33] | 6.130 | 0.047 |
| Animal welfare | 3.50 [2.75, 4.50] | 3.50 [2.50, 4.25] | 3.75 [2.50, 5.00] | 1.935 | 0.380 |
| Low fat | 4.00 [3.00, 5.00] | 4.00 [3.00, 5.00] | 4.00 [3.00, 5.00] | 2.302 | 0.316 |

*Values are presented as median [Q1, Q3]. Subscale scores were calculated as mean item scores and range from 1 to 7. Group differences were examined using the Kruskal-Wallis H test. The Bonferroni-adjusted significance threshold for the seven omnibus comparisons was p < 0.0071 (0.05/7). No subscale met this adjusted threshold. Therefore, pairwise comparisons were not interpreted.*
