# Supplementary material for: The mediating role of healthy eating attitudes in the relationship between nutrition literacy and sustainable and healthy eating behaviors among young adults: a cross-sectional study
Source: Front Public Health. 2026 Jul 8;14:1885664. doi: 10.3389/fpubh.2026.1885664 (PMC13388279; doi:10.3389/fpubh.2026.1885664)
Supplement: Supplementary file 3 [file Table_3.DOCX]

**Supplementary Table S3.** Sex differences in SHE Behaviors subscale scores

| Panel A. Unadjusted sex differences | | | | | | | | | |
| --- | --- | --- | --- | --- | --- | --- | --- | --- | --- |
| SHE Behaviors subscale | **Female median**  **[Q1, Q3]** | **Male median**  **[Q1, Q3]** | | **U** | | **Z** | ***p*** | | **r** |
| Quality labels | 3.50 [2.75, 4.13] | 3.38 [2.50, 4.34] | | 43138.0 | | -0.874 | 0.382 | | 0.036 |
| Seasonal food and avoiding food waste | 4.00 [3.00, 4.71] | 3.86 [3.00, 4.71] | | 42635.5 | | -1.112 | 0.266 | | 0.045 |
| Healthy and balanced diet | 4.00 [3.00, 5.00] | 4.00 [3.00, 5.25] | | 43421.0 | | -0.743 | 0.458 | | 0.030 |
| Local food | 3.00 [2.33, 4.00] | 3.00 [2.67, 4.67] | | 42182.0 | | -1.330 | 0.183 | | 0.054 |
| Meat reduction | 3.00 [2.33, 4.00] | 3.00 [2.33, 4.00] | | 44042.0 | | -0.450 | 0.653 | | 0.018 |
| Animal welfare | 3.25 [2.50, 4.25] | 3.75 [2.81, 4.75] | | 40209.5 | | -2.260 | 0.024 | | 0.092 |
| Low fat | 4.00 [3.00, 5.00] | 4.00 [3.00, 5.33] | | 44860.5 | | -0.062 | 0.950 | | 0.003 |
| Panel B. Adjusted sensitivity models for male sex | | | | | | | | | |
| SHE Behaviors subscale | **B** | | **Standardized beta** | | **95% CI for B** | | | ***p*** | |
| Quality labels | 0.179 | | 0.071 | | -0.005, 0.364 | | | 0.056 | |
| Seasonal food and avoiding food waste | 0.070 | | 0.027 | | -0.122, 0.262 | | | 0.475 | |
| Healthy and balanced diet | 0.217 | | 0.072 | | 0.001, 0.433 | | | 0.049 | |
| Local food | 0.304 | | 0.106 | | 0.082, 0.526 | | | **0.007** | |
| Meat reduction | 0.094 | | 0.034 | | -0.124, 0.311 | | | 0.398 | |
| Animal welfare | 0.380 | | 0.138 | | 0.168, 0.593 | | | **< 0.001** | |
| Low fat | 0.228 | | 0.078 | | 0.009, 0.448 | | | 0.042 | |

*Values in Panel A are presented as median [Q1, Q3]. Subscale scores were calculated as mean item scores and range from 1 to 7. Sex differences in Panel A were examined using the Mann–Whitney U test. Panel B presents the coefficient for male sex from separate linear regression models, each adjusted for nutrition literacy, healthy eating attitudes, food insecurity, age, and BMI, with female as the reference category. For both panels, the Bonferroni-adjusted significance threshold for the seven subscale comparisons was p < 0.0071 (0.05/7). Bold p-values denote associations meeting this threshold; p-values between 0.0071 and 0.05 are reported but were not interpreted as significant after correction. Effect size r in Panel A was calculated as the absolute Z statistic divided by the square root of N.*
