# Supplementary material for: The mediating role of healthy eating attitudes in the relationship between nutrition literacy and sustainable and healthy eating behaviors among young adults: a cross-sectional study
Source: Front Public Health. 2026 Jul 8;14:1885664. doi: 10.3389/fpubh.2026.1885664 (PMC13388279; doi:10.3389/fpubh.2026.1885664)
Supplement: Supplementary file 4 [file Table_4.DOCX]

**Supplementary Table S4.** Comparison of the main model and the FIES-adjusted sensitivity model

| **Effect** | **Main model B** | **Main model β** | **Main model 95% BC CI** | **FIES-adjusted model B** | **FIES-adjusted model β** | **FIES-adjusted model**  **95% BC CI** |
| --- | --- | --- | --- | --- | --- | --- |
| S-NutLit-Tr → ASHN | 0.254 | 0.262 | 0.181 to 0.335 | 0.247 | 0.256 | 0.176 to 0.326 |
| ASHN → SHE Behaviors | 0.675 | 0.176 | 0.380 to 0.963 | 0.678 | 0.177 | 0.383 to 0.969 |
| Direct effect | 1.739 | 0.469 | 1.376 to 2.049 | 1.745 | 0.470 | 1.381 to 2.054 |
| Indirect effect | 0.171 | 0.046 | 0.095 to 0.275 | 0.168 | 0.045 | 0.091 to 0.270 |
| Total effect | 1.911 | 0.515 | 1.571 to 2.205 | 1.913 | 0.515 | 1.572 to 2.211 |

*Values are unstandardized coefficients, standardized estimates, and bias-corrected 95% bootstrap confidence intervals. The main model was adjusted for age, sex, and BMI. The sensitivity model additionally included the total FIES score as a covariate predicting both ASHN and SHE Behaviors. All exogenous variables were allowed to covary freely. BC CI: bias-corrected confidence interval; FIES: Food Insecurity Experience Scale.*
